# Health Information Seeking Among University Students Before and During the Corona Crisis—Findings From Germany

**DOI:** 10.3389/fpubh.2020.616603

**Published:** 2021-01-25

**Authors:** Markus Schäfer, Birgit Stark, Antonia M. Werner, Ana Nanette Tibubos, Jennifer L. Reichel, Daniel Pfirrmann, Dennis Edelmann, Sebastian Heller, Lina Marie Mülder, Thomas Rigotti, Stephan Letzel, Pavel Dietz

**Affiliations:** ^1^Department of Communication, Johannes Gutenberg University Mainz, Mainz, Germany; ^2^Department of Psychosomatic Medicine and Psychotherapy, University Medical Center of the Johannes Gutenberg University Mainz, Mainz, Germany; ^3^Institute of Occupational, Social and Environmental Medicine, University Medical Center of the University of Mainz, Mainz, Germany; ^4^Department Sport Medicine, Rehabilitation and Disease Prevention, Institute of Sport Science, Johannes Gutenberg University Mainz, Mainz, Germany; ^5^Department of Work, Organizational, and Business Psychology, Institute for Psychology, Johannes Gutenberg University Mainz, Mainz, Germany; ^6^Leibniz Institute for Resilience Research, Mainz, Germany

**Keywords:** health information seeking, university students, COVID-19, SARS-CoV-2, risk perception, risk behavior

## Abstract

Health information-seeking behavior is the process of gathering information about health and disease and can be influential for health-related perception and behavior. University students are an important target group for prevention and health promotion and largely belong to an age group that is considered to play a leading role in propagating the SARS-CoV-2 epidemic in Germany. The paper deals with students' health information-seeking behavior before and during the corona crisis, aiming to give insights into its determinants and implications. Using the example of a large German comprehensive university and based on two cross-sectional surveys in the summer of 2019 (*n* = 4,351) and 2020 (*n* = 3,066), we investigate which information channels students use for health information, how information seeking changes during the course of the pandemic, and to what extent information seeking is associated with risk perception and risk behavior. For a subsample of participants that participated in both surveys (*n* = 443), we also trace developments at the individual level through a longitudinal analysis. The results show that students' health information seeking takes place primarily online and changed markedly during the corona crisis. The comparatively high relevance of sources that are largely based on unchecked user-generated content raises the concern whether students' health information-seeking behavior guarantees the necessary quality and reliability of health information. Significant correlations between the intensity of corona-related information seeking, risk perception, and actual risk behavior were found.

## Introduction

The ongoing pandemic of severe acute respiratory syndrome coronavirus 2 (SARS-CoV-2) has caused more than 300,000 detected cases of coronavirus disease 2019 (COVID-19) illness in Germany and claimed more than 9,500 lives as of October 2020 (1). While deaths related to COVID-19 are relatively rare in younger age groups in Germany, a sizable share of COVID-19-related hospitalizations also occurs in individuals of those age groups (2). Furthermore, during the first period of outbreaks in spring 2020, scholars identified that individuals aged 20 to 24 years play a leading role in propagating the SARS-CoV-2 epidemic in Germany (2), suggesting that university students, most of whom fall into this age group, play an important role in SARS-CoV-2 transmission. Additionally, in the course of the second wave of outbreaks in summer and autumn 2020, among those people infected, individuals of the younger age group were largely overrepresented (1).

Viral transmission is dependent on human behavior (3). Hence, the health-related behavior of university students is of high relevance (4, 5). The approximately three million students in Germany are, on average, 23.4 years old. More than 84% of the students are aged between 20 and 29 years old (6–8). Against the background of this age distribution, students are a particularly important target group for prevention and health promotion during the ongoing SARS-CoV-2 pandemic, given that the university setting, including teaching and learning environments with full lecture halls and changing group compositions, dormitories, and campus vibrant social life, is potentially associated with an increased risk of infection.

To address students' health risk behavior by measures of prevention and health promotion, it is especially important to assess the factors leading to this behavior, including the reasons for a possible lesser adherence to contact and physical distancing guidelines. Health information seeking and media use are considered to be such factors (9). Studies show that the way people, governments, and media communicate about health issues and health risks and how it is perceived by recipients could also be influential for perceptions, attitudes, and actual behavior in a pandemic situation (3, 10–14).

Health information-seeking behavior is the process of gathering information about health and disease (15). Representative surveys dealing with health information seeking in Germany show that media reporting is one of the most important sources of health information (16–18). These general findings are confirmed for younger age groups (9) and for the information-seeking behavior of the general population during the corona crisis (19). Between March and June 2020, 59 to 76% of the people in Germany stated that they sought information about the topic “very or rather frequently” (20) and, for this purpose, relied on mass media reporting almost daily (21).

Empirical data on the health information seeking of university students (in general as well as in particular in the corona crisis) is relevant for two main reasons: a) to identify potential health risks and health benefits of media use for this target group (and groups related to them) and b) to identify which channels are most suitable for addressing students when applying prevention and health promotion strategies. However, surveys focusing on student life and/or student health often do not take information seeking and media use into account (22). This is why rather little is known about the health information seeking of German university students in either a general or pandemic situation.

This paper investigates students' health information seeking and health-related media use before and during the corona crisis to obtain insights into German university students' behavior and its determinants and implications. Using the example of a large German comprehensive university and based on two cross-sectional surveys in the summer semesters of 2019 (*n* = 4,351) and 2020 (*n* = 3,066) as well as a longitudinal analysis of a subsample (*n* = 443) at the individual level, we investigate which channels university students use to obtain health information, how information seeking changes during the course of a pandemic situation, and to what extent information seeking is associated with risk perception and risk behavior.

## The Role of Health Information Seeking in Risk Perception

In general, people tend to claim to be less subjected to (health) risks than others, with people's risk perception being more realistic when they already have some direct or indirect experience (23). Beyond that, several other factors influence risk perception and risk behavior, such as real risk, risk sensitivity, perceived control, individual characteristics, attitudes, and media exposure (23–25). Some factors might be particularly influential for the social group of university students and/or in the context of pandemics in particular. For example, Cruwys et al. (11) assumed that shared group membership, mediated through an increased trust in other members and a reduced disgust toward these members and their behavior, leads to a decrease in risk perception and increased risk taking. Accordingly, potential threats arising from ingroup members are perceived as less risky and lead to greater risk-taking behavior than potential threats arising from outgroup members, which can consequently result in an accelerated spread of an infectious disease such as COVID-19 in the course of a pandemic (10, 11).

This general tendency could have important implications with regard to students who form a quite homogeneous group in terms of age, education, and socialization. It seems plausible that students might perceive social interactions with their peers on campus and in their private life as less risky than it actually is, potentially explaining, at least in part, why this particular group might have played a leading role in fueling the SARS-CoV-2 epidemic in Germany (2). This tendency would be in line with findings showing that young adults consider themselves comparatively invulnerable and do not think of health as an important criterion for their behavior, since they perceive themselves as healthy and have hardly any experience of illness, neither themselves nor in their peer group (26).

These tendencies could even be reinforced (or, in the better case, weakened) by a similar health information-seeking behavior within the group. In general, the need for a closer look at information seeking results from the possible effects on perceptions, attitudes, and behaviors that can emanate from the content received (27). This content can have an impact on, for example, the perception of social (health) risks and of personal (health) risks (25). Media content is particularly considered not only to influence which topics people think about and which they think are important (28), but also how recipients perceive and evaluate certain information and issues (29) and how they may act (30, 31). Following the assumptions of the cultivation theory (32–34), exposure to media content results in the formation of perceptions and beliefs about the social reality that are consistent with the media's portrayals of reality (and therefore possibly inconsistent with reality). The influence of media is greater the less recipients can rely on their experiences or the experiences of their social environment, which, on the one hand, applies to experiences of illness in young adults in general (26) and, on the other hand, applies particularly to the early stage of a pandemic when there are no reported cases in the direct social environment, especially as long as the outbreaks take place in foreign cities and countries.

Trends in general media use (19, 35), as well as findings suggesting that young people are more open to new media and technologies (15, 19, 36, 37), indicate that the health information seeking of students might differ markedly from that of other target groups. Looking at general media use in Germany in the most relevant age groups under 30, online media are by far the most important (35). There is hardly any structural difference between (daily) online users and the general population in these age groups. Furthermore, a representative survey of German Internet users on health information seeking shows that online media is used to a greater extent by the relevant age groups for health-related purposes (9). Social media seems to be relatively important as health-related information sources in general and for the coronavirus in particular, especially in younger age groups (19). In light of international findings showing a high relevance of online information sources among college students (15), it is therefore highly likely that for German university students in particular, online media might be an important source of health information.

Surveys of the general population show that media use changed significantly during the corona crisis (19). Health-relevant effects of media use on perceptions, attitudes, and behavioral intentions have also been reported in the context of pandemics. In the ongoing SARS-CoV-2 pandemic, researchers found that patients more frequently described symptoms of a loss of smell or taste when media had previously reported on them (38). During the H1N1 pandemic 2009/2010, for example, a lack of trust in media reporting resulting from a perceived low quality of media coverage was associated with lower vaccination intentions (39). Not only but even more particularly during pandemics, media use is therefore considered to have a more or less important influence on health-related risk perception and risk behavior (12, 14, 24, 25, 40).

Considering the potential important role of university students as a target group for health prevention and health promotion in general as well as during the ongoing pandemic in particular, the possible changes of information seeking during a pandemic crisis, and the possible influences of information seeking for risk perception and risk behavior, the present study asks:

1) Which channels do university students use for health information?
2) How does information seeking change in the course of a pandemic situation?
3) To what extent is information seeking associated with risk perception and risk behavior during a pandemic situation?

## Methods

To address students' information-seeking behavior and its implications for risk perception and risk behavior during the corona crisis, we conducted two online surveys in summer 2019 and 2020 at a large German comprehensive university (about 31,500 students) with a full range of disciplines and subjects, located in a German mid-size city (about 210,000 inhabitants). Both surveys were conducted as part of an ongoing evidence-based student health initiative in a 2 year interval, which involves detailed surveys of the student body regarding important health-related factors (around 270 items, mostly validated standard instruments and partly self-designed or adapted items). However, since living and study conditions suddenly changed so dramatically during the pandemic (and resulted in the abovementioned research questions), we conducted an additional survey within this acute pandemic situation amending self-designed COVID-related items.

Recruitment was accomplished in both cases by an email that was sent to all students currently enrolled at the university. Students received this email in their accounts where they normally received important notifications (e.g., about their grades). The emails were tailored to the target group and emphasized different monetary and non-monetary incentives to increase motivation for participation. Reminder emails were sent at different times.

Participants had to be enrolled in at least one subject of study at the local university. Answering at least one relevant question regarding relevant health variables such as health information seeking (in addition to demographic variables) was a prerequisite to be included for further analyses.

Approval to perform the studies was obtained by the ethical committees of the Medical Association of Rhineland-Palatinate (study I: No. 2019-14336) and the Institute of Psychology of Johannes Gutenberg University Mainz (study II: No. 2020-JGU-psychEK-S008). Both studies were designed as cross-sectional surveys. Nevertheless, the repeated measurement of certain items over time allows at least for some participants, to some extent, statements not only on a macro level but also on an individual level, as at the beginning of each survey the respondents created an individualized unique anonymous code that allowed a comparison of the results for respondents participating in both surveys. For a subsample of students that participated in both surveys (*n* = 443), we could therefore trace certain developments at an individual level.

We surveyed the frequency of health information seeking and the sources and channels used for this purpose. To measure the frequency of information seeking, participants were asked how frequently they have “sought information on health and disease in the past 12 months?” (study I) and “sought information on this topic in recent weeks?” (study II), respectively. Answer options were “never,” “less than once a month,” “at least once a month,” “at least once a week,” and “at least once a day.” Additionally, we recorded the frequency of information seeking in days per week (0–7; less than at least once a week = 0; at least once a day = 7; participants who stated to have sought information at least once a week were asked to indicate the exact number of days per week).

To record the sources of information, we asked the participants where they have sought information “on health and disease in the past 12 months” (study I) and “on the coronavirus during recent weeks” (study II), respectively. In a first step, participants could indicate whether they have used certain information sources (e.g., interpersonal sources, e.g., family members, friends, and colleagues; health professionals; pharmacists; other patients; mass media sources, e.g., offline mass media, online media) or not. Multiple answers were possible. In a second step, participants who indicated to have used online sources were asked which online sources they have used “for information on health and diseases” (study I) and “to obtain information about the coronavirus?” (study II), respectively. Participants could indicate whether they have used certain online sources (e.g., websites of health organizations, doctors or health insurance companies, journalistic online news sites, blogs on health and disease, social media, video platforms, online encyclopedia like Wikipedia or search engines like Google) or not. Again, multiple answers were possible.

As possible determinants, we surveyed different demographics such as age, gender, the intended degree as well as health interest, health literacy, and health status (e.g., perceived health condition, presence of a chronic disease) of students. Health interest was measured on a five-point Likert scale from “not interested at all” to “very interested”^1^. Health literacy was recorded on a four-point scale (“very easy,” “fairly easy,” “fairly difficult,” “very difficult”) with the help of a short version of the of the German Health Literacy Questionnaire (HLS-GER), including four items [“How easy/difficult would you say it is to…” a) “find information about symptoms of illnesses that concern you?”; b) “understand what to do in a medical emergency?”; c) “judge when you need to go to a doctor for a check-up?”; and d) “make decisions to improve your health?”; sum score including all four single items: 0–12]. Participants rated their perceived health condition on a scale ranging from 0 = “worst conceivable state of health” to 10 = “best conceivable state of health.” They further indicated if they have been diagnosed with a chronic disease or not.

The questionnaire of the second survey included additional questions on risk perception, risk behavior, general vaccination motivation, and specific vaccination intentions that could be influential during a pandemic situation. Regarding risk perception, we asked the students to indicate on a seven-point scale from “not at all likely” to “absolutely likely” how likely it was in their opinion that within a 2 month time frame (a) they get infected with the coronavirus; (b) their family members, friends, and colleagues get infected with the coronavirus; (c) they need hospitalization in case of an infection; (d) they are quarantined, regardless of an infection; and (e) they get infected and infect others with the virus. To measure risk behavior, participants indicated if they did comply with the five central recommendations of health authorities (to wash hands often and intensely; to use antiseptics; to reduce meetings and personal contacts; to wear a mask; to avoid crowded places) in the week before the survey or not. A sum score (0–5) was calculated. General vaccination motivation was recorded by a single item [“How important is it for you to have sufficient vaccination protection against common diseases (e.g., mumps, measles, rubella, tetanus)?”] on a five-point Likert scale from “not at all important” to “very important.” We further asked the students how likely they would get vaccinated if a vaccine against the coronavirus was available. They could scale their answer on an 11-point Likert scale from “very unlikely” to “very likely.”

Study I was conducted in June and July 2019 during a regular semester^2^ with classroom teaching and physical presence on the university campus. In total, 4,351 university students participated in the survey, demonstrating a response rate of 13.9% of the total student population at that time. Compared with the distribution of the university, female and younger students were overrepresented in the sample (Table 1).

**Table 1:** Mean age and distribution of gender in the sample, the university student population, and the student population in Germany in percent.

	**Study I** ** (*n* = 4,351)**	**Study II** ** (*n* = 3,066)**	**Subsample I/II** ** (*n* = 443)**	**University** ** (*n* = 31,500)**	**Germany** ** (*n* = 2.9 million)**
Age	23.8 (mean)	23.4 (mean)	22.8/23.7 (mean)	24.7 (mean)	23.4 (median)
**Gender**					
Male	28.6	26.8	22.6	41.0	51.1
Female	70.5	72.6	77.0	59.0	48.9
Diverse	0.9	0.7	0.5	–	–

Of the participants, 52% were studying for a bachelor's degree and 21.1% for a master's degree. Another 22.5% were aiming for a German state exam (e.g., law and medical students, students of teaching professions), and 3.4% were Ph.D. students. While 92% of the participants were born in Germany, 24% had at least one parent with a migration history.

The participants were highly interested in the topic of health and disease and considered their own health status to be relatively good (Table 2). Female participants were slightly more interested in health and disease and reported a slightly lower health status than male participants. Participants who identified themselves as diverse stated a significantly lower interest in the topic and a significantly poorer health condition.

**Table 2:** Interest in health and perceived health status of the participants by gender.

**Measure**	**Total**	**Male**	**Female**	**Diverse**
Interest in health and disease^a^	4.0	3.9	4.1	3.5
Perceived health status^b^	7.4	7.5	7.4	6.3

*n = 4,351*.

a *Question: “Some people are particularly interested in the topic of health and disease, others not at all. What about you? How interested are you in the topic?” (1 = not at all interested; 5 = very interested)*.

b Question: “If you rate the best conceivable state of health with a 10 and the worst conceivable state of health with a 0, how many points do you give for your current state of health?”

The existence of SARS-CoV-2 was first confirmed in China at the end of December 2019, and there are indications of a spread within Europe from December 2019 (41). In Germany, the first cases of COVID-19 diseases were reported in January 2020. By mid-July 2020, Germany detected almost 200,000 confirmed cases (42). At that time, the share of hospitalizations among the detected cases was about 17%, and the share of deaths was 4.6% (42, 43). In consequence, during the summer semester of 2020, the life and work of university students in Germany were heavily affected by the first outbreaks of the novel coronavirus and the following emergency situation. The acute pandemic situation resulted in far-reaching measures regarding social and university life to contain the epidemic and a shutdown of nearly all university facilities in Germany right before the start of the summer term in April 2020. Emergency operation was guaranteed at most German universities and resulted in the exceptional situation of almost exclusively online teaching during the summer semester without any physical presence or social activities at the universities allowed.

Study II was conducted in June and July 2020, at the heart of this pandemic situation. Overall, 3,066 students participated in our survey, demonstrating a response rate of 9.7% of the total student population at that time. The sociodemographic structure of the sample was like that of the first study. Therefore, 56% of the participants were aiming for a bachelor's degree, 21% for a master's degree, 21.6% were aiming for a German state exam (e.g., law and medical students, students of teaching professions), and 0.4% were Ph.D. students. Compared with the distribution of the university, female and younger students were again overrepresented in the sample (Table 1).

There were some notable differences regarding students' rooming situation between study I and study II that came along with the pandemic. At the time of study II, 37.1% lived with their parents or other relatives (study I: 21.6%), 10.5% lived in a student dormitory (study I: 15.7%), another 11.3% lived alone in an apartment (study I: 15.5%), 20.2% shared their apartment with their partner and/or children (study I: 19.7%), and 20.9% lived with roommates of a shared apartment (study I: 27.4%). Of the students in our sample, 0.5% indicated to have already been infected with the coronavirus.

For a subsample of students that participated in both surveys (*n* = 443), we traced developments at the individual level. Those students were significantly younger at the time of the first survey compared with the general sample of study I (Table 1); this was unsurprising because students' age increases as the time of study graduation approaches, which increases the probability for younger students and decreases the probability for older students that they (can) participate in both studies. At the time of the second survey (about) 1 year later, the age distribution in the subsample consequently reflected approximately the age distribution in the main samples of study I and study II. Female students were even more overrepresented in the subsample, accounting for more than three-quarters of all respondents. However, there were no differences regarding general interest in health and disease between the main and subsamples.

All statistical analyses were performed with IBM SPSS, version 23.

## Results

### Study I

The students in our sample were quite active with regard to their health information-seeking behavior. Of the students, 80% stated that they sought information on health and disease at least once a month, 41% stated that they engaged in health information seeking every week, and 11% every day. Only 1% of the students indicated that they did not seek health information during that respective period, and 19% stated that they sought information less regularly than once a month (Table 3).

**Table 3:** Frequency of health information seeking.

**Frequency of information seeking**	**Study I** ** %**	**Study II** ** %**
Never	1.2	0.6
Less than once a month	19.3	4.0
At least once a month	38.5	12.6
At least once a week	30.2	54.6
At least once a day	10.9	28.1

χ^2^ = 1,309.48, p < 0.001; study I: n = 4,347; Question: “How frequently have you sought information on health and disease in the past 12 months?”; study II: n = 2,994; Question: “Some people seek information on the coronavirus on a daily basis, while others never seek such information. We are interested in how you deal with it. How frequently have you sought information on this topic in recent weeks?”

On average, students sought information on health and disease 1.5 days per week (SD = 2.4). Female students (*M* = 1.6; SD = 2.4) were slightly more active than male students (*M* = 1.4; SD = 2.3) and students who identified themselves as diverse (*M* = 1.2; SD = 2.3), but without statistically significant differences (*F* = 1.94; *p* > 0.05). Bachelor students (*M* = 0.9; SD = 1.6) showed significantly less intensive health-seeking behavior (days per week) than master students (*M* = 1.1; SD = 1.9), Ph.D. students (M = 1.6; SD = 2.6), and students aiming for a German state exam (*M* = 3.4; SD = 3.0) (*F* = 153.85; *p* < 0.001). Students with chronic diseases (*M* = 2.0; SD = 2.5) sought health information significantly more often than students without such diseases (*M* = 1.4; SD = 2.3) (*t* = 6.62; *p* < 0.001).

General interest in health and disease was positively associated with the intensity of health information seeking (*r* = 0.48; *p* < 0.001), while no such correlation was evident between the perceived health status and health information-seeking behavior (*r* = 0.01; *p* > 0.05). Health literacy was positively associated with the intensity of health information seeking (*r* = 0.25; *p* < 0.001).

Online sources were by far the most important information sources on health and disease for the students^3^. More than 90% reported that they used online media for health information seeking during the 12 months preceding the study. Also quite important were interpersonal contacts with family members, friends, and colleagues (75%) and health professionals (59%). By contrast, the reporting of offline mass media (40%) and personal contacts with pharmacists (20%) or other patients (13%) played a rather subordinate role as sources of health information.

**Figure 1:**
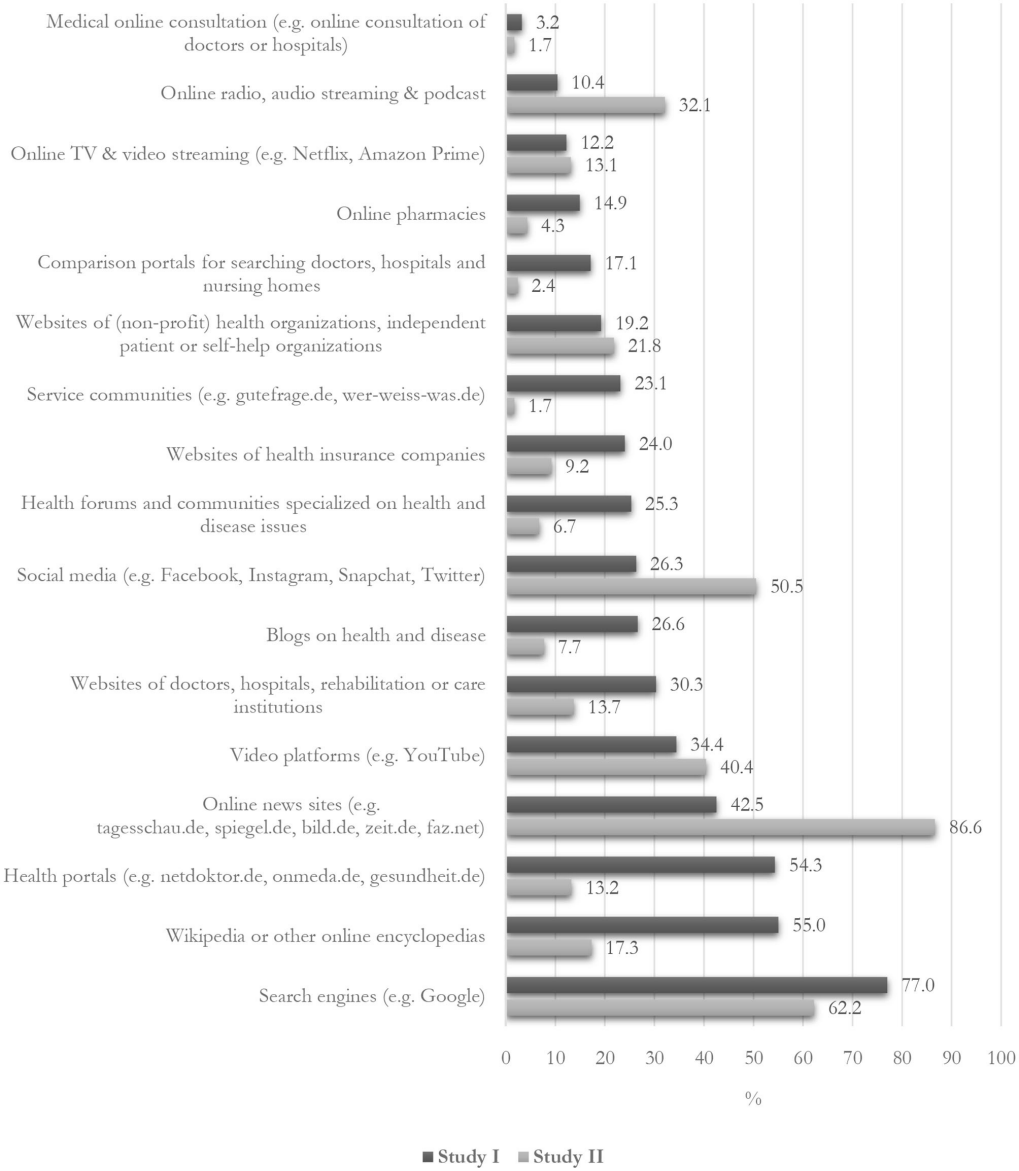
Online sources for information on health and disease (study I) and on coronavirus (study II), respectively. Percentage among participants. Study I: *n* = 3,920; Question: “Which online sources have you used in the past 12 months for information on health and diseases?”; study II: *n* = 2,735; Question: “Which online sources have you used to obtain information about the coronavirus?”; Pearson's chi-square: *medical online consultation*: χ^2^ = 14.20, *p* < 0.001; *online radio, audio streaming, and podcast*: χ^2^ = 484.99, *p* < 0.001; *online TV and video streaming*: χ^2^ = 1.02, *p* > 0.05; *online pharmacies*: χ^2^ = 191.92, *p* < 0.001; *comparison portals*: χ^2^ = 355.07, *p* < 0.001; *websites of (non-profit) health organizations, independent patient or self-help organizations*: χ^2^ = 6.51, *p* < 0.05; *service communities*: χ^2^ = 603.55, *p* < 0.001; *websites of health insurance companies:* χ^2^ = 238.89, *p* < 0.001; *health forums and communities specialized on health and disease issues:* χ^2^ = 384.15, *p* < 0.001; *social media*: χ^2^ = 405.95, *p* < 0.001; *blogs on health and disease*: χ^2^ = 376.12, *p* < 0.001; *websites of doctors, hospitals, rehabilitation, or care institutions*: χ^2^ = 244.40, *p* < 0.001; *video platforms*: χ^2^ = 24.91, *p* < 0.001; *online news sites*: χ^2^ = 1,314.98, *p* < 0.001; *health portals*: χ^2^ = 1,161.57, *p* < 0.001; *Wikipedia or other online encyclopedias*: χ^2^ = 955.23, *p* < 0.001; *search engines*: χ^2^ = 169.54, *p* < 0.001.

Looking at the concrete online sources that students, who utilized online media for health information seeking, used for information on health and disease, our data show that students accessed health information often *via* search engines (77%; Figure 1). The most relevant providers for students' online information on health and disease included Wikipedia or other online encyclopedias (55%), specialized health portals (54%), online news sites (43%), and video platforms like YouTube (34%). Websites of health professionals and health institutions, special blogs on health and disease, social media and health forums, and online forums were frequented by one in four to one in three of the students, while medical online consultations played almost no role.

### Study II

The intensity of students' health-related information seeking increased markedly during the corona crisis. More than 95% of the respondents reported that they sought information on the coronavirus at least once a month, 83% stated that they sought information at least every week, and 28% sought information every day. Only 0.6% of the participants indicated that they did not seek information about the coronavirus, and 4% stated that they sought information less regularly than once a month (Table 3).

On average, students sought information on the coronavirus 3.6 days per week (SD = 2.6). The intensity of information seeking did not differ significantly between students with (*M* = 3.7; SD = 2.6) and without (*M* = 3.6; SD = 2.6) chronic diseases (*t* = 1.39; *p* > 0.05), as it could have been supposed in consequence of a possible perception to be more vulnerable due to potential risk factors for a severe course of the disease. As in study I, no correlation was evident between perceived health status and information seeking (*r* = 0.03; *p* > 0.05). Health literacy was not positively associated with the intensity of information seeking either (*r* = 0.03; *p* > 0.05), but with the perceived health status (*r* = 0.18; *p* < 0.001). As in study I, no significant differences (*F* = 0.51; *p* > 0.5) were evident between female students (*M* = 3.6; SD = 2.6), male students (*M* = 3.7; SD = 2.6), and students who identified themselves as diverse (*M* = 3.4; SD = 2.7).

Compared with students' general health information-seeking behavior beyond a pandemic situation, interpersonal contacts with family members, friends, and colleagues (81%) became even more important to the students as a source of information during the corona crisis^4^. Notably, the same applied to classic offline mass media reporting (68%), while personal contacts with health professionals (19%), pharmacists (4%), or other patients (4%) were of little importance.

In addition, during the corona crisis, online sources were by far the most important information sources for the students (92%). However, again, decisive shifts in concrete online media use in the context of an acute pandemic situation could be observed (Figure 1). On the one hand, there was a clear tendency toward a greater use of journalistic news media. Almost 87% of the students indicated that they had utilized journalistic online news websites for information seeking. Search engines were highly relevant to access information on the coronavirus (62%) but were not used as widely as for health information seeking in general. On the other hand, social media played a much more important role regarding information seeking during the corona crisis. More than half of the students stated that they used social media services, such as Facebook, Instagram, Snapchat, and Twitter, for information seeking. The students also used video platforms (40%), online radio, audio streaming, and podcasts (32%), as well as websites of health organizations (22%), such as the WHO or the Robert Koch Institute (RKI), more intensively. By contrast, online encyclopedias (17%), blogs (8%), and online health forums (7%) played a relatively minor role in students' information seeking on the coronavirus.

Significant correlations existed between the intensity of corona-related information seeking and risk perception and actual risk behavior (Table 4). A higher/lower extent of corona-related information seeking was associated with a higher/lower perceived susceptibility to get infected with the new coronavirus and a higher/lower perceived likelihood of needing hospitalization, of infecting others with the virus in case of an infection, of being quarantined regardless of an infection, and of persons in the closer social environment getting infected. In addition, a higher/lower extent of corona-related information seeking went along with a higher/lower compliance with recommendations aimed at containing the spread of the virus (e.g., washing hands often and intensely, using antiseptics, reducing meetings and personal contacts, wearing a mask, and avoiding crowded places), as well as with a higher/lower intention to get vaccinated if a vaccine against the coronavirus were available.

**Table 4:** Correlations between the intensity of information seeking on the coronavirus and corona-related risk perception, risk behavior, and vaccination intentions.

**Measure**	***r***
Risk perception	
Perceived likelihood of getting infected with the coronavirus	0.12^***^
Perceived likelihood of family members, friends, and colleagues • getting infected with the coronavirus	0.10^***^
Perceived likelihood of needing hospitalization in case of infection	0.06^**^
Perceived likelihood of being quarantined, regardless of infection	0.13^***^
Perceived likelihood of getting infected and infecting others with • the virus	0.07^***^
Risk behavior and vaccination intentions	
Compliance with recommendations^a^	0.17^***^
Vaccination intentions against coronavirus^b^	0.16^***^

Pearson's correlation; n = 2,987–2,991;

** p < 0.01;

*** *p < 0.001*.

a *Compliance with recommendations to wash hands often and intensely, use antiseptics, reduce meetings and personal contacts, wear a mask, and avoid crowded places in the week before the survey*.

b Question: “How likely would get vaccinated if a vaccine against the coronavirus were available?”

### Subsample: Individual Level

Relevant changes in students' information-seeking behavior were also found in the subsample on an individual level, which underlines the validity of the observed changes based on the cross-sectional data of study I and study II (Table 5). Very similar shifts can be observed. In the first survey, while 19.7% of the students stated that they sought information on health and disease never or less regularly than once a month, only 3.9% maintained this behavior during the corona crisis. Instead, almost 85% stated that they sought information on the coronavirus at least every week, and one-third every day, an increase of 47 and 26 percentage points compared with general health information seeking, respectively. The average frequency of information seeking among the students increased from 1.3 (SD = 2.1) to 4.0 days (SD = 2.6) a week, with statistically significant differences (*t* = 18.48; *p* < 0.001). The intensity of general health information seeking and corona-related information seeking was only moderately correlated (*r* = 0.21; *p* < 0.001), indicating that, although there are certain tendencies related to general habits of the students, many students showed substantial changes in their behavior in the pandemic situation.

**Table 5:** Changes in frequency of health information seeking in the subsample (individual level, T1 and T2).

**Frequency of information seeking**	**T1 (study I)** ** %**	**T2 (study II)** ** %**
Never	0.7	0.2
Less than once a month	19.0	3.7
At least once a month	42.2	11.2
At least once a week	31.2	51.6
At least once a day	7.0	33.3

Subsample: students who participated in both surveys; n = 443; Question T1: “How frequently have you sought information on health and diseases in the past 12 months?”; Question T2: “Some people seek information on the coronavirus on a daily basis, while others never seek such information. We are interested in how you deal with it. How frequently have you sought information on this topic in recent weeks?”

The average number of information sources and online information sources used for health-related information seeking varied significantly between the time before and during the corona crisis. While the students used 3.9 different information sources for general health information (SD = 1.7), for corona-related information, they used only 3.2 different sources on average (SD = 1.3; *t* = 8.76; *p* < 0.001). A similar decline was observed for the number of online health information sources, which dropped from an average of 5.3 different sources (SD = 2.7) to 4.0 (SD = 2.1; *t* = 8.93; *p* < 0.001), indicating a more focused use of (certain health-related) information sources during the pandemic.

Different from the cross-sectional sample, there were no or only very small significant correlations regarding the intensity of information seeking, risk perception, and actual risk behavior. A higher/lower extent of corona-related information seeking was slightly associated with a higher/lower perceived likelihood of persons in the closer social environment getting infected (*r* = 0.10; *p* < 0.05), a higher/lower tendency to wash hands often and intensely (*r* = 0.11; *p* < 0.05), a higher/lower general vaccination motivation (*r* = 0.15; *p* < 0.01), and a higher/lower intention to get vaccinated if a vaccine against the coronavirus was available (*r* = 0.15; *p* < 0.01). General health information-seeking behavior (T1) was slightly associated with the perceived likelihood of being quarantined later on (T2; *r* = 0.10; *p* < 0.05). However, in contrast to our cross-sectional data, no other significant correlations between information seeking and risk perception respective risk behavior were found.

## Discussion

Our data show that student health information seeking takes place primarily online. The comparatively high relevance of search engines and sources, which are largely based on user-generated content, raises the question of whether students' health information-seeking behavior guarantees the necessary quality and reliability of health information, knowing that wrong and erroneous information can lead to serious health-related consequences. In our view, there is a need for further research in this area on the one hand, and a clear potential for education campaigns in the university context that focus on the quality and seriousness of online health information and students' health literacy on the other. In turn, for health professionals and health authorities that deal with students' health and health behavior, these findings make clear that it makes sense not to ignore but instead actively use these communication channels, adapt their messages accordingly to the channels and target groups, and provide reliable information where students actually seek for information.

In particular, against the background of the ongoing corona pandemic, these aspects are of central importance. We can see that in the acute pandemic situation, students' health-related information-seeking behavior shifted significantly. First, the intensity of information seeking increased markedly, while the number of information sources used decreased significantly. Second, students' focus was much more on original journalistic news sources, interpersonal sources, and social media. The reduced diversity of information sources and the comparatively lower importance of search engines could indicate that content from known (and maybe trusted) sources were used in a much more targeted manner. Nevertheless, interpersonal contacts and the great importance of social media and video platforms like YouTube bear the danger of fake news and misinformation that can be especially dangerous in a pandemic situation. At the same time, our data show that a comparatively small percentage of students used information from primary sources, such as health organizations or health professionals, during the pandemic.

For health authorities interested in addressing students' health behavior by means of communication, these findings offer both opportunities and challenges. On the one hand, the increased intensity of information seeking shows a general need and openness for information that trusted sources could satisfy and address by reaching out important health messages to the target group. On the other hand, the reduced diversity of students' information sources and the rather low importance of primary sources clearly restricts the choice of communication channels for this aim during a pandemic situation. Health communication professionals must definitely be aware of these conditions and adapt and tailor their actions to them if they want to be heard.

We found significant correlations with very low (almost negligible) to low-effect sizes between the intensity of corona-related information seeking, risk perception, and actual risk behavior in our cross-sectional data, indicating the possible importance of health information seeking as a potential influencing factor on perception and behavior during the pandemic. At the same time, no such constant correlations with risk perception and risk behavior were found for general health information seeking or corona-related information seeking in the significantly smaller subsample, which could indicate (but does not necessarily mean) that the significant interrelations found might be mainly due to sample size. Accordingly, the findings should be interpreted with care. Furthermore, the direction of the observed relationship is unclear, with both directions being plausible and the possibility of variables influencing each other. It seems highly plausible that a perceived higher risk of being infected can result in a perceived higher need for further information on the topic. At the same time, given the high general relevance of health information for risk perception, the presentation of a (higher/lower) risk by certain information sources could lead to a higher/lower risk perception of the recipients. Likewise, the observed (higher/lower) compliance with health recommendations could be either a result of certain types of risk presentation or, for example, just the expression of certain personality traits leading to a certain information-seeking behavior. Although, based on theoretical assumptions and earlier findings, it can be assumed that information-seeking behavior can have consequences for risk perception and risk behavior, further research is definitively needed.

Although we found that information seeking and risk perception during the pandemic are associated, this association seems to be less strong than one might assume. This could indicate that the information that students receive through the various information channels (and here in particular the mass media reporting that is highly relevant as a source of information in the corona crisis) does not necessarily lead to an increased risk awareness among students. This, in turn, could be related to the widespread narrative, which is also quite common in media coverage (but questionable in terms of content), according to which the protective measures are primarily necessary for protecting the elderly and vulnerable groups. This could lead to the (false) perception among students (and other young people) that the risk of infection for themselves is rather unlikely.

In fact, students tended to estimate the risk of infecting themselves with the virus as rather low. Only 12% of the students considered the risk of infection to be rather, very, or absolutely likely. This is interesting because the age distribution of the infections in most countries suggests that although older people have a greater risk of getting seriously ill, the risk of infection seems to be quite independent of age. In fact, especially during the summer months, in Germany, a disproportionately high number of young people were infected with the virus.

Personal health status does not seem to be associated with the intensity of health information seeking, neither before nor during the pandemic. In contrast, the general interest in health and disease—and this relationship—seems to be quite strong. The same applies for health literacy. This could indicate that even students who perceive their own health condition to be poor might not engage in getting further (and maybe highly relevant) information if they are not interested in health and disease in general and have a rather low health literacy. For health authorities that are especially interested in sending relevant health messages to vulnerable groups that suffer from bad health conditions, it could therefore make sense to also engage in broader campaigns to first increase students' general health literacy and general interest in the topic of health and disease.

Given the great relevance of social media, video platforms such as YouTube, and search engines as sources of health information for students in general and in an acute pandemic situation, the characteristics and quality of the concrete content presented by these information sources and used by the students must be given greater attention in future research. Providers of such platforms should be aware that they have a great responsibility. Fake news and misinformation must be monitored and rigorously pursued and prevented by content moderation and quality monitoring. At the same time, it is important to strengthen the media and health literacy of students to empower their competence to judge which sources and information they can rely on and which they cannot.

However, it should not be overlooked that, especially in a concrete pandemic situation, journalistic news sources, regardless of how they are accessed, still seem to be particularly important for young people to obtain reliable health information. Journalistic reporting is thus highly relevant in the corona crisis from a students' perspective, a tendency that has also been observed in the general population. This finding should incentivize journalistic media to maintain and improve the quality of reporting. At the same time, the question arises as to which concrete sources of information students use and how this use differs from other groups of the population. More in-depth investigations would be necessary, particularly qualitative studies.

As a first step in this line of inquiry, our empirical study has limitations that future work may overcome. First, our samples are structurally different from the student population at the university investigated. As participation was voluntary, it is highly likely that health-interested students tended to participate to a much larger degree than students with a generally lower interest in health and disease, suggesting that our data perhaps missed out a target group highly relevant for health prevention and health promotion. Second, for our larger samples, we provided only cross-sectional data on a macro level. Individual trends over time could only be tracked for a comparatively small subsample, which structurally does not fully reflect the composition of the cross-sectional samples. Further research is therefore needed.

Although we provide data for a large (and in many ways typical) German university, our investigation took place only at one university in Germany. Accordingly, the structural composition of our sample differs in some points from the group of students in Germany as a whole, although this applies less to age than to gender composition. Therefore, the generalization of the findings is limited. This is even more true in the international arena, where not only education and university systems but also the type and intensity of measures and restrictions during the pandemic can vary greatly from country to country.

Nevertheless, our study provides important clues on students' health information-seeking behavior before and during a pandemic situation that can help shape future concepts of prevention and health promotion in the university setting. Our general findings on the important role of online media for student health information seeking are largely in line with the findings of similar studies in other countries. Furthermore, it could be at least assumed that if the tendencies previously stated showed for highly educated and health-interested students, it could be likely to be even more so for less health-interested students and young people from other educational backgrounds. With regard to the specific situation in the pandemic, at this time, there is not enough reliable data available to determine to what extent the present findings are similar or different to developments in other countries. Comparative studies would be very interesting here.

Besides the necessary efforts to increase students' general health literacy, health-related media literacy, including education about the quality of certain information sources, is a central aspect that has to be addressed in the future. This includes workshop concepts to improve students' health-related search (engine) literacy as well as their assessment of the credibility of news and health information sources. Both aspects should be implemented in the context of student health management and could be implemented in school and university teaching.

## Data Availability Statement

The datasets presented in this article are not readily available because of an ongoing project. If required, data can be provided after the end of the project. Requests to access the datasets should be directed to Pavel Dietz, pdietz@uni-mainz.de.

## Ethics Statement

The studies involving human participants were reviewed and approved by Ethical committees of the Medical Association of Rhineland-Palatinate (No. 2019-14336) and the Institute of Psychology of Johannes Gutenberg University Mainz (No. 2020-JGU-psychEK-S008). The patients/participants provided their written informed consent to participate in this study.

## Author Contributions

All authors listed have made a substantial, direct and intellectual contribution to the work, and approved it for publication.

## Conflict of Interest

The authors declare that the research was conducted in the absence of any commercial or financial relationships that could be construed as a potential conflict of interest.

## Footnotes

^1^Question: “Some people are particularly interested in the topic of health and disease, others not at all. How about you: How interested are you in the topic?”

^2^The academic year at most German universities is divided in two terms: a winter semester, usually starting mid-October, and a summer semester, starting in mid-April.

^3^*N* = 4,345; Question: “Where have you sought information on health and disease in the past 12 months?”

^4^*N* = 2,991; Question: “Where have you sought information on the coronavirus during recent weeks?”
